# Prevalence of Partially Hydrogenated Oils in US Packaged Foods, 2012

**DOI:** 10.5888/pcd11.140161

**Published:** 2014-08-28

**Authors:** Jenifer Clapp, Christine J. Curtis, Ann E. Middleton, Gail P. Goldstein

**Affiliations:** Author Affiliations: Christine J. Curtis, Ann E. Middleton, Gail P. Goldstein, New York City Department of Health and Mental Hygiene, Queens, New York.

## Abstract

Although there is evidence that consumption of trans fat has declined in the United States, limited documentation exists on current levels of industrial trans fat in foods. We estimated the prevalence of partially hydrogenated oils in 4,340 top-selling US packaged foods. Nine percent of products in the sample contained partially hydrogenated oils; 84% of these products listed “0 grams” of trans fat per serving, potentially leading consumers to underestimate their trans fat consumption. Government efforts to eliminate partially hydrogenated oils from packaged foods will substantially reduce exposure to this known cardiovascular disease risk factor.

## Objective

Trans fat consumption is a risk factor for cardiovascular disease (1). The US Food and Drug Administration (FDA) has tentatively determined that partially hydrogenated oils (PHOs), the main dietary source of industrial trans fat, are not “generally recognized as safe” for consumption (2). The FDA is considering public comments on this determination. If FDA finalizes the proposed change, products containing PHOs will not be allowed as ingredients in packaged or restaurant food unless the FDA makes a determination that they are safe. This study estimates the prevalence of PHOs in US packaged foods to better understand the implications of the proposed restriction of PHOs.

## Methods

To estimate the prevalence of industrial trans fat in the packaged food supply, we used a cross-sectional database of brand-name products developed for the National Salt Reduction Initiative (NSRI) in 2012 (3). The NSRI Packaged Food Database (NSRI database) includes products in 61 commonly consumed food categories including baked goods, frozen foods, and snacks (4) that represent many of the top contributors of dietary trans fat (5). The NSRI database contains all products in the top 80% of sales for 61 food categories in 2011, a total of 8,024 products. Nutrition label and ingredient information for these products was purchased from Guiding Stars Licensing Company in January 2012 and supplemented with data collected from manufacturer websites, outreach to manufacturers, and visits to supermarkets from January through June 2012. Although some foods contain naturally occurring trans fat derived from small amounts in the byproducts of ruminant animals, most dietary trans fat comes from PHOs. Because manufacturers are permitted to label products containing between 0 and 0.5 g of trans fat per serving as “0 grams” in the United States, we identified products that contained PHOs by the presence of the words “partially hydrogenated” in the ingredient list. Trans fat label data and ingredient information were available for 4,340 products, which make up the sample for this analysis.

The number of products with PHOs and mean trans fat per serving were calculated using SAS version 9.2 (SAS Institute Inc). Serving size was not standardized.

## Results

Of the 4,340 products with trans fat label and ingredient data, 391 (9%) listed PHOs in their ingredient information. Of those, 61 products (16%) reported trans fat content per serving in excess of 0 grams or 0.5 grams or more per serving (mean = 1.66; 95% confidence interval, 1.38–1.92; range, 0.5–4.5). The balance of products with PHOs in the ingredient information, 330 products (84%), listed trans fat as 0 grams per serving on the Nutrition Facts label. The amount of trans fat in these products could vary from trace amounts to almost 0.5 g of trans fat per serving.

The Figure shows the number of products in each food category with PHOs, stratified by the reported amount of product trans fat per serving (0 g or ≥0.5 g). Products containing PHOs make up 50% of products in the seasoned processed potatoes category (15 of 30) and 35% of products in the cookies category (76 of 218). Over half of the NSRI food categories include at least 1 product with PHOs, including but not limited to many types of baked goods and snack foods (eg cookies, crackers, frozen entrees and sides served in less than 6-ounce servings).

**Figure:**
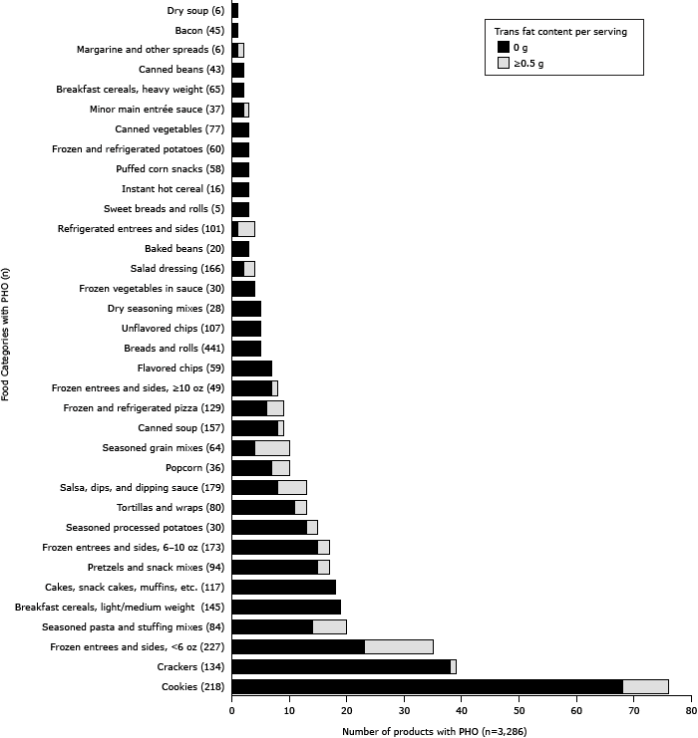
Number of products made with partially hydrogenated oils, by National Salt Reduction Initiative food category, 2012 (n = 35) **Table:** 

Category Name and Number of Products in the Category	Number of Products With Trans Fat	
Food Category (n)	0g	≥0.5g
Cookies (218)	68	8
Crackers (134)	38	1
Frozen entrees & sides, <6 oz (227)	23	12
Seasoned pasta and stuffing mixes (84)	14	6
Breakfast cereals, light or medium weight (145)	19	0
Cakes, snack cakes, muffins, etc. (117)	18	0
Pretzels and snack mixes (94)	15	2
Frozen entrees and sides, 6–10 oz (173)	15	2
Seasoned processed potatoes (30)	13	2
Tortillas and wraps (80)	11	2
Salsa, dips, and dipping sauce (179)	8	5
Popcorn (36)	7	3
Seasoned grain mixes (64)	4	6
Canned soup (157)	8	1
Frozen and refrigerated pizza (129)	6	3
Frozen entrees and sides, ≥10 oz (49)	7	1
Flavored chips (59)	7	0
Breads and rolls (441)	5	0
Unflavored chips (107)	5	0
Dry seasoning mixes (28)	5	0
Frozen vegetables in sauce (30)	4	0
Salad dressing (166)	2	2
Baked beans (20)	3	0
Refrigerated entrees and sides (101)	1	3
Sweet breads and rolls (5)	3	0
Instant hot cereal (16)	3	0
Puffed corn snacks (58)	3	0
Frozen and refrigerated potatoes (60)	3	0
Canned vegetables (77)	3	0
Minor main entrée sauce (37)	2	1
Breakfast cereals, heavy weight (65)	2	0
Canned beans (43)	2	0
Margarine and other spreads (6)	1	1
Bacon (45)	1	0
Dry soup (6)	1	0

In food categories with at least 1 product containing PHOs (35 categories, 3,286 products), an average of 15% of products per category contain PHOs, ranging from 0.4% in bacon to 66% in seasoned processed potatoes. In 2011, sales for these products totaled $3.5 billion.

## Discussion

Our analysis demonstrates that industrial trans fat is still common in US packaged foods, particularly in some food categories. These findings, which are consistent with FDA research findings (6), provide evidence of the prevalence of industrial trans fat and show that most products that contain PHOs are labeled as containing 0 g of trans fat (84%). This labeling is cause for concern because consumers, seeing the 0 g trans fat on the Nutrition Facts label, are probably unaware that they are consuming trans fat. Comparable PHO-free products were available in every food category assessed and make up 50% or more of products in all categories with PHOs, indicating that removing PHOs from packaged foods is feasible. Our study has some limitations, primarily related to limited availability of nutrition and ingredient data and the unavailability of restaurant data.

Eliminating trans fat from US foods is possible, but removal has not been achieved through labeling requirements for packaged food: almost 1 in 10 products we examined contained PHOs. Although restricting the use of PHOs in packaged food would benefit consumers preparing foods at home, an FDA ruling would also help ensure that restaurant customers are protected from unknowingly consuming industrial trans fat. Some local jurisdictions have restricted the use of PHOs in food service establishments, but most Americans live in areas where no such regulation exists. Scientific evidence shows that even low levels of trans fat intake pose a risk to consumers (7). Because of the FDA’s current labeling requirements, people continue to unknowingly consume PHOs.
